# Vasorelaxant effect of *Prunus yedoensis* bark

**DOI:** 10.1186/1472-6882-13-31

**Published:** 2013-02-14

**Authors:** Kyungjin Lee, Inhye Ham, Gabsik Yang, Mihwa Lee, Youngmin Bu, Hocheol Kim, Ho-Young Choi

**Affiliations:** 1 Department of Herbology, College of Korean Medicine, Kyung Hee University, 1 Hoegi-Dong, Dongdaemun-Gu, 130–701, Seoul, Republic of Korea

## Abstract

**Background:**

*Prunus yedoensis* Matsum. is used as traditional medicine—‘Yaeng-Pi’ or ‘Hua-Pi’—in Japan and Korea. However, no studies have examined the pharmacological activities of the *P. yedoensis* bark. Only the antioxidant and antiviral activities of *P. yedoensis* fruit and the anti-hyperglycaemic effect of *P. yedoensis* leaf have been investigated. While studying the antihypertensive effects of several medicinal plants, we found that a methanol extract of *P. yedoensis* bark (MEPY) had distinct vasorelaxant effects on rat aortic rings.

**Methods:**

The aortic rings were removed from Sprague–Dawley rats and suspended in organ chambers containing 10 ml Krebs-Henseleit solution. The aortic rings were placed between 2 tungsten stirrups and connected to an isometric force transducer. Changes in tension were recorded via isometric transducers connected to a data acquisition system.

**Results:**

MEPY relaxed the contraction induced by phenylephrine (PE) both in endothelium-intact and endothelium-denuded aortic rings concentration dependently. However, the vasorelaxant effects of MEPY on endothelium-denuded aortic rings were lower than endothelium-intact aortic rings. The vasorelaxant effects of MEPY on endothelium-intact aortic rings were reduced by pre-treatment with l-NAME, methylene blue, or ODQ. However, pre-treatment with indomethacin, atropine, glibenclamide, tetraethylammonium, or 4-aminopyridine had no affection. In addition, MEPY inhibited the contraction induced by extracellular Ca^2+^ in endothelium-denuded rat thoracic aorta rings pre-contracted by PE (1 μM) or KCl (60 mM) in Ca^2+^-free solution.

**Conclusions:**

Our results suggest that MEPY exerts its vasorelaxant effects via the activation of NO formation by means of l-Arg and NO-cGMP pathways and via the blockage of extracellular Ca^2+^ channels.

## Background

*Prunus yedoensis* Matsum (PY) is the most popular and widely cultivated cherry tree in Japan and Korea [1,2]. The bark of PY has been used in traditional medicine -‘Yaeng-Pi’ or ‘Hua-Pi’ [3]- to treat cough, urticaria, pruritus, dermatitis [4], asthma, and measles [3]. However, no studies have examined the pharmacological activities of PY bark. Only the antioxidant and antiviral activities of PY fruit [5] and the anti-hyperglycaemic effect of PY leaf [6] have been investigated.

While conducting an *in vitro* screening study of various medicinal plants of vasorelaxant effect on the isolated rat thoracic aorta rings using organ chamber technique, PY bark was found to exhibit distinct vasorelaxant activity. The effects of PY bark on the vascular system have not been studied previously. Therefore, the present study was designed to examine the vasorelaxant effect of a methanol extract of PY bark (MEPY) on isolated rat thoracic aortic rings.

## Methods

### Plant material and extraction

*P. yedoensis* bark was purchased from Dongwoodang Co., Ltd. (Yeongcheon, Kyungpook, Republic of Korea) in June 2007. Professor Hocheol Kim of Kyung Hee University identified the plants. A voucher specimen *P. yedoensis* bark (PY001) was deposited at the College of Korean Medicine, Kyung Hee University, Seoul, Republic of Korea. Dried *P. yedoensis* bark (3 kg) was extracted 3 times with 100% MeOH for 3 h in a reflux apparatus. After reflux and filtration, the extract was evaporated using a rotary vacuum evaporator (N-N series, EYELA, Japan) at 60°C and lyophilized to yield 386.8 g of crude extract. MEPY (1 g) was dissolved in dimethyl sulfoxide (DMSO; 10 ml).

### Chemicals and drugs

Phenylephrine (PE), acetylcholine (ACh), *N*_ω_-Nitro-L-arginine methyl ester (l-NAME), methylene blue (MB), atropine, indomethacin, ethylene glycol-bis (β-aminoethyl ether)-N,N,N’,N’-tetraacetic acid (EGTA), tetraethylammonium (TEA), glibenclamide, 4-aminopyridine (4-AP), 1-H-[1,2,4]-oxadiazolo-[4,3-α]-quinoxalin-1-one (ODQ), and DMSO were purchased from Sigma Aldrich (St. Louis, USA). All other reagents were of analytical purity.

### Preparation of rat aortic rings

All procedures involving animals were conducted according to the animal welfare guidelines issued by National Veterinary Research & Quarantine Service and World Organization for Animal Health (OIE); and this study was approved (KHUASP(SE)-10–028) by the Kyung Hee University Institutional Animal Care and Use Committee. The rats were housed under controlled conditions (22 ± 2°C; lighting, 07:00–19:00), and food and water were available ad libitum. Sprague–Dawley rats (weight, 240–260 g; Narabio, Seoul, Korea) were anesthetized by exposure to ether, and the thoracic aorta was removed and immersed in Krebs-Henseleit solution [K-H solution, composition (mM): NaCl, 118.0; KCl, 4.7; MgSO_4_, 1.2; KH_2_PO_4_, 1.2; CaCl_2_, 2.5; NaHCO_3_, 25.0; and glucose, 11.1; pH 7.4], maintained at 37°C, and aerated with a mixture of 95% O_2_ and 5% CO_2_. After connective tissue and fat were carefully removed, approximately 2-mm-long aortic rings were cut and suspended in organ chambers containing 10 ml K-H solution at 37°C. The rings in the chambers were aerated with a mixture of 95% O_2_ and 5% CO_2_. The aortic rings were placed between 2 tungsten stirrups and connected to an isometric force transducer (Grass Instrument Co., Rhode Island, USA). After incubation under no tension for 30 min, the vessel segments were allowed to equilibrate for 1 h at a resting tension of 1.0 g. The K-H solution was replaced every 15 min during the equilibration period. Changes in tension were recorded via isometric transducers connected to a data acquisition system (PowerLab, ADI instrument Co., New South Wales, Australia). When required, the endothelium was removed by gently rubbing the lumen of the vessel with a thin cotton swab. The presence of functional endothelium was verified by the ability of ACh (10 μM) to induce more than 80% relaxation in rings that were precontracted by PE (1 μM). In endothelium-denuded rings, ACh caused less than 10% relaxation. Ca^2+^-free K-H solution was prepared by replacing CaCl_2_ with EGTA (1 mM).

### Vasoactivity

We studied the concentration-dependent relaxant effect of MEPY on endothelium-intact and endothelium-denuded aortic rings that were pre-contracted with PE (1 μM) in standard K-H solution. The relaxant effect of MEPY on the aortic rings was calculated as a percentage of the contraction in response to PE.

To study the effect of MEPY on nitric oxide (NO) synthesis pathway, endothelium-intact aortic rings were pre-incubated with l-NAME (10 μM) for 20 min before contraction by PE (1 μM) treatment. The relaxant effect of MEPY on the aortic rings was compared with the control (not treated with l-NAME).

To investigate the effect of MEPY on the NO-cyclic guanosine monophosphate (cGMP) pathway, endothelium-intact aortic rings were pre-incubated with ODQ (10 μM) or MB (10 μM) for 20 min before contraction by PE (1 μM) treatment. The relaxant effect of MEPY on the aortic rings was compared with the control (not treated with ODQ or MB).

To determine whether prostacyclin is involved in MEPY-induced vasorelaxation, endothelium-intact aortic rings were pre-incubated with indomethacin (1 μM) for 20 min before contraction by PE (1 μM) treatment. The relaxant effect of MEPY on the aortic rings was compared with the control (not treated with indomethacin).

To determine whether enhanced NO release by MEPY was associated with the activation of muscarinic receptors, endothelium-intact aortic rings were pre-incubated with atropine (1 μM) for 20 min before contraction by PE (1 μM) treatment. The relaxant effect of MEPY on the aortic rings was compared with the control (not treated with atropine).

To determine whether K^+^ channels are involved in MEPY-induced vasorelaxation, endothelium-intact aortic rings in standard K-H solution were pre-incubated with various K^+^ channel blockers, TEA (5 mM), glybenclamide (10 μM), or 4-AP (1 mM) for 20 min before the addition of PE (1 μM). Once a plateau was attained, MEPY (1–100 μg/ml) was cumulatively added. The vasorelaxant effect of MEPY on the aortic rings was calculated as a percentage of the contraction in response to PE.

To investigate the effect of MEPY on extracellular Ca^2+^-induced contractions, we carried out 2 sets of experiments: (1) evaluation of receptor-operative Ca^2+^ channels (ROCCs) and (2) evaluation of voltage-dependent Ca^2+^ channels (VDCCs). In the experiment on ROCCs, we investigated the contractile response induced by CaCl_2_ (0.3–10 mM) in the endothelium-denuded aortic rings contracted by PE (1 μM) in Ca^2+^-free K-H solution with and without (control) a 10-min preincubation with MEPY (200 μg/ml). The experiment on VDCCs followed the same procedure, except that the contraction was induced by KCl (60 mM). The contractile responses induced by CaCl_2_ in the presence and absence (control) of MEPY pre-treatment were compared.

### Data analysis

Data are expressed as mean ± standard error of mean (SEM). Statistical comparisons were made using Student’s t-test or one-way analysis of variance (ANOVA) followed by the Tukey’s post-hoc test. All statistical analyses were performed using SPSS v.13.0 statistical analysis software (SPSS Inc., USA). P values less than 0.05 were considered statistically significant.

## Results

### Effect of MEPY on PE-induced contraction of endothelium-intact or endothelium-denuded aortic rings

**Figure 1:**
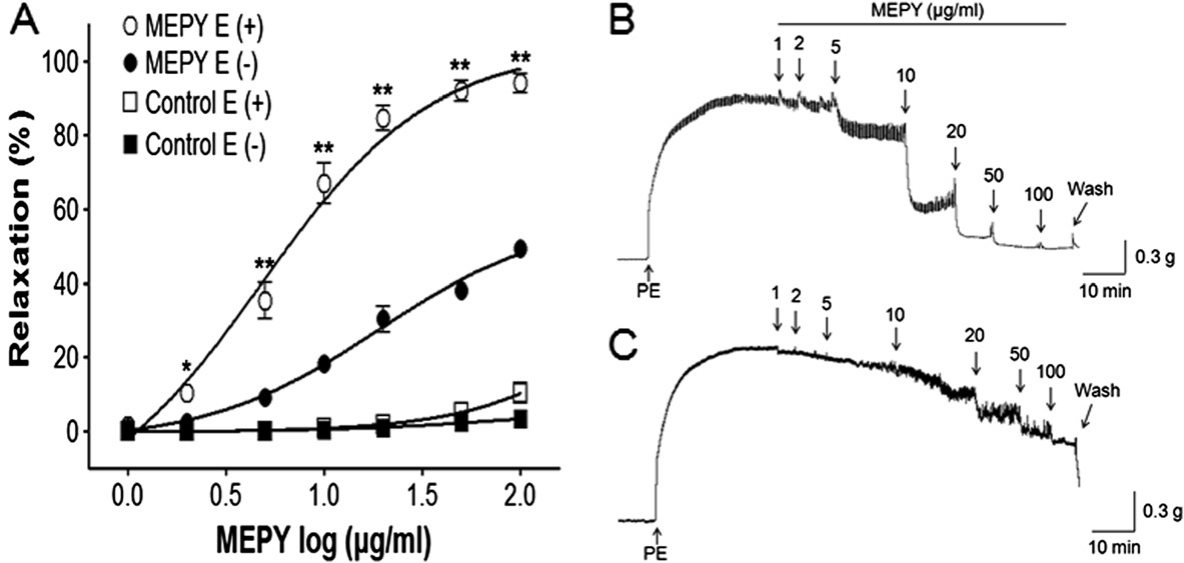
**Concentration-dependent relaxant effects of MEPY on phenylephrine (PE, 1 μM)-pre-contracted rat aortic rings with [(E+)] or without [(E-)] endothelium (A) in Krebs-Henseleit solution.** Control groups were not treated with MEPY. The MEPY induced-relaxant traces of aortic rings with [(E+)] **(B)** or without [(E-)] endothelium **(C)**. The relaxant effects of MEPY on isolated rat aortic rings were calculated as a percentage of the contraction in response to PE. Values are expressed as mean ± SEM (n = 8). ^*^P < 0.05, ^**^P < 0.01 *vs*. MEPY E (−).

MEPY caused concentration-dependent relaxation in both endothelium-intact and endothelium-denuded aortic rings pre-contracted by PE (1 μM) treatment. However, endothelium-intact aortic rings were more relaxed than endothelium-denuded aortic rings. The maximal relaxant effect was 94.0% ± 2.7% and 49.4% ± 0.7% for endothelium-intact and endothelium-denuded aortic rings, respectively (Figure 1).

### Effect of MEPY on endothelium-intact aortic rings pre-incubated with l-NAME, MB, or ODQ

**Figure 2:**
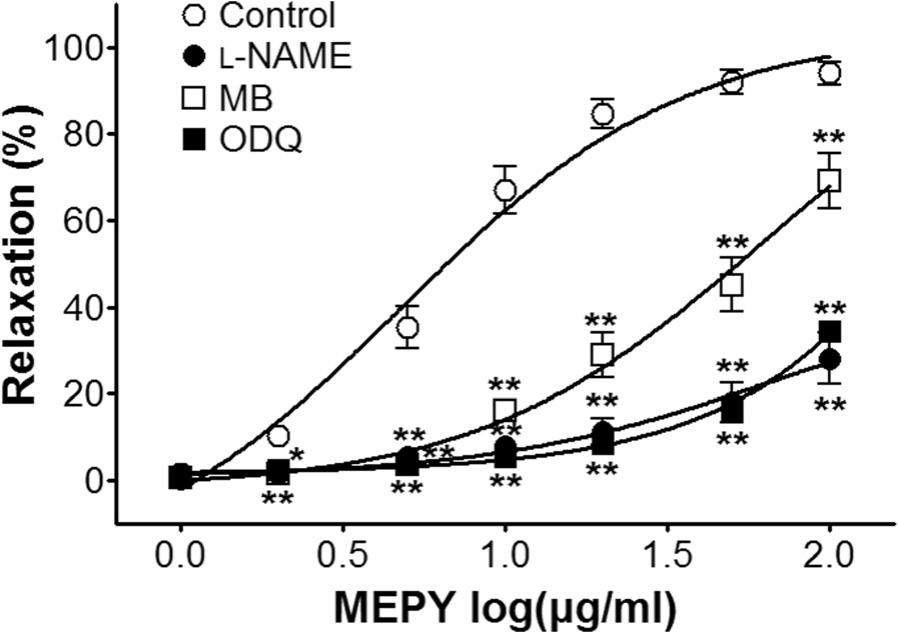
**Relaxation responses induced by MEPY in endothelium-intact rat aortic rings pre-contracted with phenylephrine (PE, 1 μM) in the presence or absence (control) of *N***_**ω **_**-Nitro-L-arginine methyl ester (l-NAME, 10 μM), methylene blue (MB, 10 μM), or 1-H-[1,2,4]-oxadiazolo-[4,3-α]-quinoxalin-1-one (ODQ, 10 μM) in Krebs-Henseleit solution.** The relaxant effects of MEPY on isolated rat aortic rings were calculated as a percentage of the contraction in response to PE. Values are expressed as mean ± SEM (n = 6–8). ^*^P < 0.05, ^**^P < 0.01 *vs*. control.

Incubation with l-NAME (10 μM), MB (10 μM), or ODQ (10 μM) significantly decreased MEPY-induced relaxation of endothelium-intact aortic rings pre-contracted by PE (1 μM) treatment. In the absence of l-NAME, MB, or ODQ, the maximal relaxant effect was 94.0% ± 2.7%. In the presence of l-NAME, MB, and ODQ, the maximal relaxant effect was 28.1% ± 5.9%, 69.2% ± 6.5%, and 34.5% ± 0.6%, respectively (Figure 2).

### Effect of MEPY on endothelium-intact aortic rings pre-incubated with indomethacin or atropine

**Figure 3:**
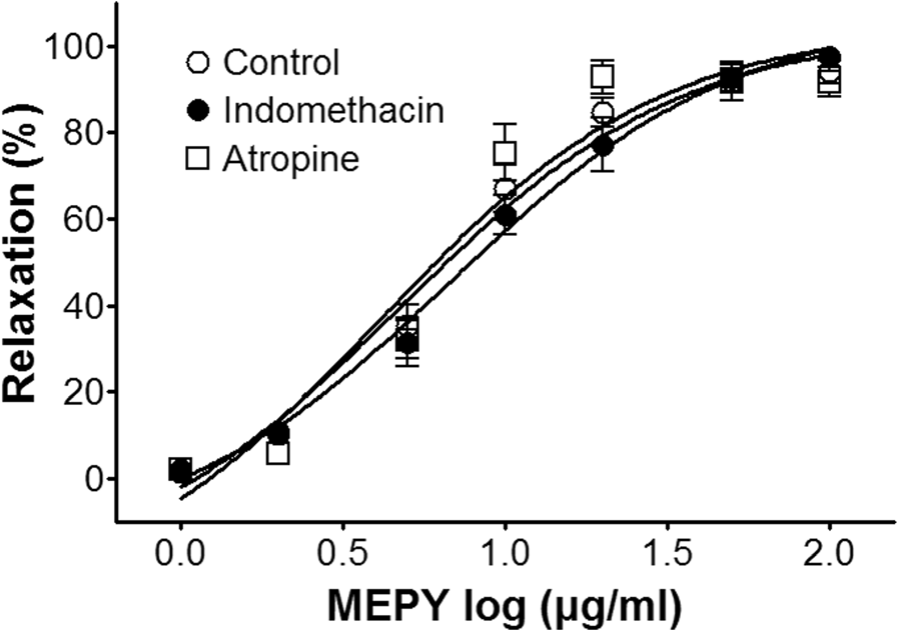
**Relaxation responses induced by MEPY in endothelium-intact rat aortic rings pre-contracted with phenylephrine (PE, 1 μM) in the presence or absence (control) of indomethacin (1 μM) or atropine (1 μM) in Krebs-Henseleit solution.** The relaxant effects of MEPY on isolated rat aortic rings were calculated as a percentage of the contraction in response to PE. Values are expressed as mean ± SEM (n = 4–8).

Incubation with indomethacin (1 μM), a non-selective cyclooxygenase (COX) inhibitor, did not affect MEPY-induced relaxation of endothelium-intact aortic rings pre-contracted by PE (1 μM) treatment (Figure 3).

### Effect of MEPY on endothelium-intact aortic rings pre-incubated with various K^+^ channel blockers

**Figure 4:**
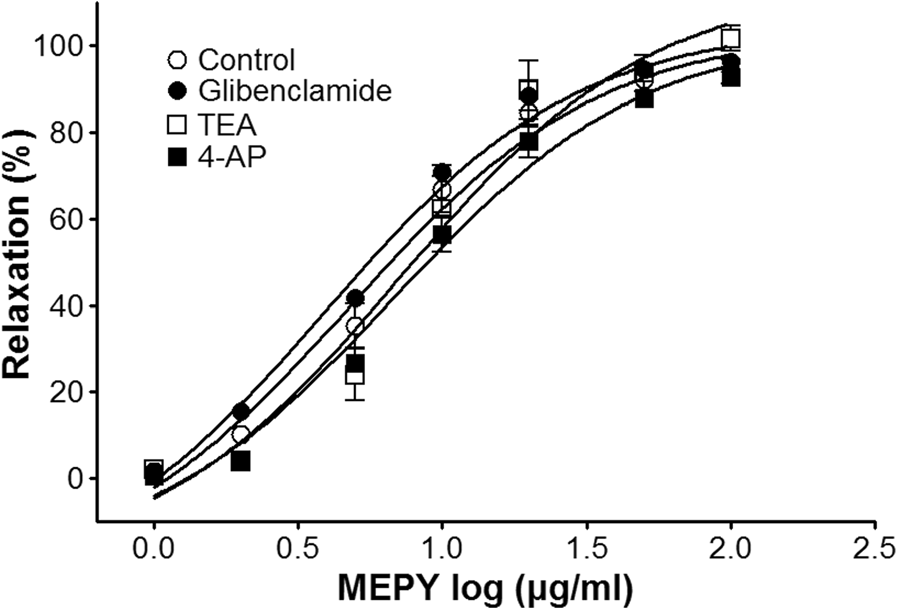
**Relaxation responses induced by MEPY in endothelium-intact rat aortic rings pre-contracted with phenylephrine (PE, 1 μM) in the presence or absence (control) of glibenclamide (10 μM), tetraethylammonium (TEA, 5 mM), or 4-aminopyridine (4-AP, 1 mM) in Krebs-Henseleit solution.** The relaxant effects of MEPY on isolated rat aortic rings were calculated as a percentage of the contraction in response to PE. Values are expressed as mean ± SEM (n = 6–8).

The vasorelaxant effects of MEPY on PE (1 μM)-pre-contracted endothelium-intact aortic rings were not altered by incubation of the rings with various K^+^ channel blockers, including glibenclamide (10 μM), tetraethylammonium (TEA, 5 mM), or 4-aminopyridine (4-AP, 1 mM) (Figure 4).

### Effect of MEPY on extracellular Ca^2+^-induced contraction

**Figure 5:**
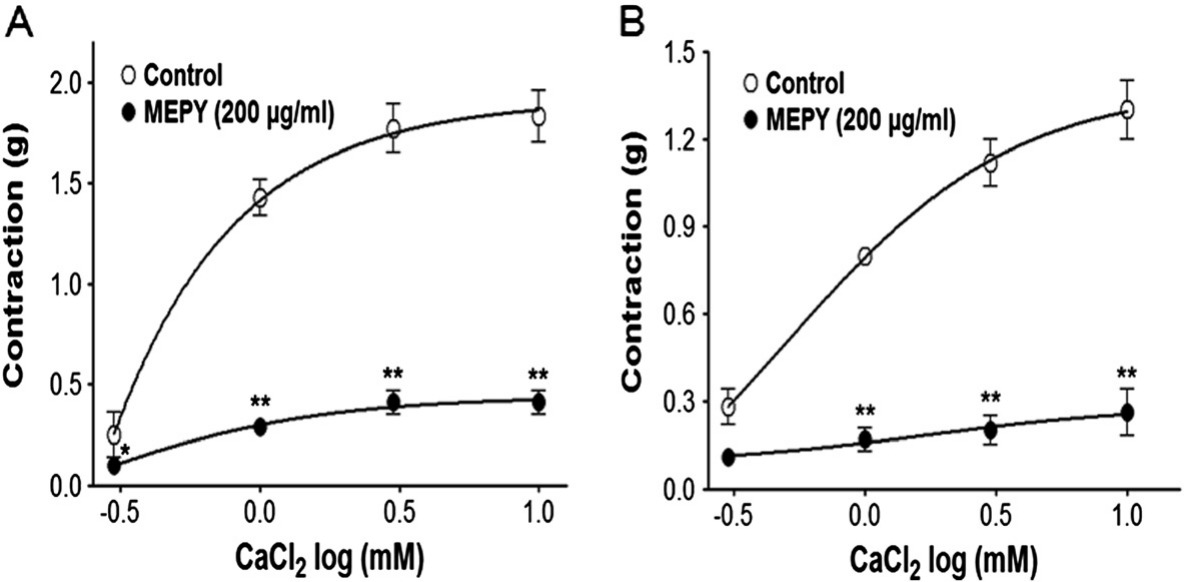
**Inhibitory effect of MEPY (200 μg/ml) on the contraction induced by extracellular Ca **^**2+**^**addition (0.3–10 mM) in endothelium-denuded rat aortic rings pre-contracted by phenylephrine (PE, 1 μM) (A) or KCl (60 mM) (B) in Ca**^**2+**^**-free Krebs-Henseleit solution.** Values are expressed as mean ± SEM (n = 5–7). ^*^P < 0.05, ^**^P < 0.01 *vs*. control.

In Ca^2+^-free K–H solution, the cumulative addition of CaCl_2_ (0.3–10 mM) induced progressively increased tension in the rat aortic rings pre-contracted by PE (1 μM; Figure 5A) or KCl (60 mM; Figure 5B) treatment. As shown in Figure 5, MEPY (200 μg/ml) pre-incubation for 20 min significantly inhibited the contraction induced by extracellular CaCl_2_.

## Discussion

In the present study, MEPY showed relaxant effects on both PE- and KCl-pre-contracted endothelium-intact and endothelium-denuded aortic rings. However, the relaxant effects of MEPY on endothelium-denuded aortic rings were reduced. These findings suggested that the relaxant effects caused by MEPY were both endothelium-dependent and endothelium-independent. Thus, we clarified the mechanisms of MEPY on rat aortic rings through endothelium dependent pathway in addition to endothelium independent pathway. Thus, the main findings were as follows: (1) the vasorelaxant effect of MEPY was related to activation of nitric oxide (NO) formation from l-arginine (l-Arg) and the NO-cGMP pathway; (2) vasoactive prostacyclin (PGI_2_) and muscarinic receptors and K^+^ channels did not contribute to MEPY-induced vasorelaxation; (3) MEPY relaxed the aortic rings by blockage of the entry of extracellular Ca^2+^ via ROCCs and VDCCs.

The vascular endothelium plays an important role in vasorelaxation via the secretion of potent vasodilators such as NO, PGI_2_, and endothelium-derived hyperpolarizing factor (EDHF). In endothelial cells, the calcium-calmodulin complex stimulates NO synthase (NOS), which activates NO formation from l-Arg. In the smooth muscle cells, NO stimulates soluble guanylate cyclase, which increases intracellular cGMP; this increase then activates cGMP-dependent protein kinases leading to a decrease in the calcium concentrations in the smooth muscle cells. Vascular smooth muscle is relaxed via these pathways [7].

To investigate the involvement of endothelium-derived vasodilators, the effects of various inhibitors of MEPY-induced relaxation were examined. The vasorelaxant effect of MEPY was reduced by pre-treatment with l-NAME, an inhibitor of NOS. Moreover, pre-treatment with MB or ODQ, which are soluble guanylate cyclase inhibitors, reduced the vasorelaxant effects of MEPY. These findings suggested that the vasorelaxant effect of MEPY is related to activation of NO formation from l-arginine and NO-cGMP pathway.

However, pre-treatment with indomethacin, a non-selective cyclooxygenase (COX) inhibitor did not affect the vasorelaxant effects of MEPY. PGI_2_ is synthesized by COX in the endothelial cells, and it activates adenyl cyclase (AC). It causes an increase in the intracellular concentrations of cyclic adenosine monophosphate (cAMP), which leads to vasodilation by decreasing intracellular calcium concentrations in the vascular smooth muscle [7]. This finding suggested that PGI_2_ may not contribute to MEPY-induced vasorelaxation.

In addition, pre-treatment with atropine, a non-selective muscarinic receptor antagonist did not affect the vasorelaxant effects of MEPY. This suggested that MEPY did not interact with the muscarinic receptors in MEPY-induced vasorelaxation.

The vasorelaxant effects of MEPY were not affected by pre-treatment with various K^+^ channel blockers. The opening of K^+^ channels in vascular smooth muscle cells is a major mechanism involved in the regulation of muscle contractility and vascular tone [8]. TEA (a blocker of large conductance Ca^2+^-activated K^+^ channels), glybenclamide (a selective inhibitor of ATP-sensitive K^+^ channels), and 4-AP (a suppressant of voltage-gated K^+^ channels) are well-known K^+^ channel blockers used to investigate potential K^+^ channel-related vasorelaxant effects of various agents. Our findings indicated that the relaxant effect of MEPY on the rat aortic rings is not related to the opening of K^+^ channels.

In the present study, removal of the functional endothelium and pre-treatment with l-NAME reduced but did not abolish the relaxant effects of MEPY. Therefore, we concluded that MEPY-induced vasorelaxation also involves endothelium-independent pathway. Vascular smooth muscles play an important role in vasorelaxation, which is regulated by the influx of extracellular Ca^2+^ via transmembrane Ca^2+^ channels [9]. The influx of extracellular Ca^2+^ is mainly regulated by ROCCs or VDCCs [10]. MEPY (200 μg/ml) inhibited vasoconstriction induced by Ca^2+^ supplementation in the aortic rings that had been pre-contracted with PE (1 μM) in Ca^2+^-free K–H solution, suggesting that MEPY inhibits the entry of extracellular Ca^2+^ via ROCCs activated by PE. And, MEPY also inhibited the vasocontraction induced by Ca^2+^ supplementation in the aortic rings that had been pre-contracted with KCl (60 mM) in Ca^2+^-free K–H solution, suggesting that MEPY inhibits the entry of extracellular Ca^2+^ via VDCCs activated by KCl.

## Conclusions

Our findings suggested that vasorelaxant mechanisms responsible for the hypotensive effects of MEPY include endothelium-dependent and endothelium-independent pathways involving activation of NO formation from l-Arg and NO-cGMP pathway, blocking the entry of extracellular Ca^2+^ via ROCCs and VDCCs. These findings may explain the medicinal use of *P. yedoensis* bark in hypertension. However, more detailed mechanism studies, *in vivo* studies and isolation of the potent vasorelaxant single compound from MEPY may be necessary to establish the precise efficacy of MEPY on hypertension.

## Competing interests

The authors declare that they have no competing interests.

## Authors’ contributions

KL, GY, and ML participated in the experimental studies and helped to draft the manuscript. IH performed the statistical analysis. HK identified the plant. KL and YB participated in the writing of the manuscript. YB and HK participated in the editing of the manuscript. HC conceived of the study, and participated in its design and coordination and helped to draft the manuscript. All authors read and approved the final manuscript.

## Pre-publication history

The pre-publication history for this paper can be accessed here:

http://www.biomedcentral.com/1472-6882/13/31/prepub
